# Supplementary material for: Laboratory Test Results in Patients with Workplace Moisture Damage Associated Symptoms—The SAMDAW Study
Source: Healthcare (Basel). 2023 Mar 29;11(7):971. doi: 10.3390/healthcare11070971 (PMC10093791; doi:10.3390/healthcare11070971)
Supplement: Supplementary file 1 [file healthcare-11-00971-s001.zip › healthcare-2232662-supplementary.pdf]

Supplementary data

Table S1. Blood count results among study patients (N=99) and controls (N=48).

	Range			Mean		
	**Normal range**	**Patients**	**Controls**	**Patients**	**Controls**	**p**
Hemoglobin, g/L						
Women	117-155	116-148	110-160	134	133	0.495
Men	134-167	132-162	134-171	151	150	0.852
Hematocrit, proportion						
Women	0.35-0.46	0.35-0.44	0.35-0.50	0.40	0.41	0.223
Men	0.39-0.50	0.39-0.47	0.39-0.53	0.43	0.45	0.347
Erythrocyte count ($x10^{12}/L$)						
Women*	3.9-5.2	3.9-5.1	3.5-5.4	4.5	4.5	0.958
Men*	4.3-5.7	4.5-5.5	4.5-5.8	5.0	5.0	0.776
Mean corpuscular hemoglobin, pg	27-33	27-35	25-33	30	30	0.623
Mean corpuscular volume, fL	82-98	81-100	82-100	89	91	0.001
Thrombocyte count ($\times 10^9/L$)	150-360	159-382	120-374	271	257	0.136
Total leucocyte count ($\times 10^9/L$)	3.4-8.2	2.7-14.9	2.7-9.1	6.5	5.6	<0.001
Neutrophils ($\times 10^9/L$)	1.60-6.20	1.37-10.21	1.26-5.69	3.77	3.08	0.001
Eosinophils ($\times 10^9/L$)	0.01-0.45	0.02-1.01	0.01-0.76	0.14*	0.14*	0.823
Basophils ($\times 10^9/L$)	0.01-0.09	0-0.15	0.02-0.11	0.03*	0.04*	0.096
Monocytes ($\times 10^9/L$)	0.20-0.80	0.16–1.36	0.23–0.83	0.47	0.45	0.323
Lymphocytes ($\times 10^9/L$)	1.20–3.50	0.81–7.13	0.94–3.25	2.08	1.85	0.046

* Median

Table S2. Perceived stress and laboratory tests among the study patients.

	Range	Stress Low (n=73)	High (n=25)	p
Leucocytes*	<3.4	1.4%	0	0.527
	3.4-8.2	83.6%	76%	
	>8.2	15.1%	24%	
Neutrophils*	<1.60	2.8%	4.0%	1.000
	1.60-6.20	90.3%	88%	
	>6.20	6.9%	8.0%	
Eosinophils*	<0.15	58.4%	44%	0.610
	0.15-0.30	32.9%	44%	
	>0.30	12.3%	12.0%	
Lymphocytes*	<1.20	5.6%	8.0%	0.368
	1.20-3.50	91.7%	84.0%	
	>3.50	2.8%	8.0%	
Monocytes*	<0.20	0	4.0%	0.016
	0.20-0.80	100%	88%	
	>0.80	0	8.0%	
FeNO ppb	<25	68.5%	76.0%	0.218
	25-50	28.8%	16.0%	
	>50	2.7%	8.0%	
ESR mm/h	0-30	98.6%	96.0%	0.447
	>30	1.4%	4.0%	
Total IgE kU/L	0-100	86.3%	76.0%	0.229
	>100	13.7%	24.0%	
CRP mg/L	0-3	72.6%	80.0%	0.464
	>3	27.4%	20.0%	

* × 10^9/L

Table S3. Laboratory test results among patients and controls with MCS.

	Range	Patients (%)(n=39)	Controls (%)(n=5)	p
Leucocytes*	<3.4	0	20.0	0.161
	3.4-8.2	79.5	60.0	
	>8.2	20.5	20.0	
Neutrophils*	<1.60	0	20.0	0.146
	1.60-6.20	92.1	80.0	
	>6.20	7.9	0	
Eosinophils*	<0.15	41.0	60.0	0.830
	0.15-0.30	38.5	20.0	
	>0.30	20.5	20.0	
Lymphocytes*	<1.20	2.6	20.0	0.316
	1.20-3.50	94.7	80.0	
	>3.50	2.6	0	
Monocytes*	<0.20	0	0	1.000
	0.20-0.80	97.4	100	
	>0.80	2.6	0	
FeNO ppb	<25	69.2	80.0	0.135
	25-50	28.2	0	
	>50	2.6	20.0	
ESR mm/h	0-30	100%	100%	N.A.
	>30	0	0	
Total IgE kU/L	0-100	79.5	60.0	0.317
	>100	20.5	40.0	

* × 10^9/L

Table S4. Laboratory test results among the study patients scoring high in all QEESI© subscales vs. others.

	Range	All high (%) (n=26)	Others (%) (n=73)	p
Leucocytes*	<3.4	0	1.4	0.541
	3.4-8.2	76.9	83.6	
	>8.2	23.1	15.1	
Neutrophils*	<1.60	0	4.1	0.860
	1.60-6.20	92.0	89.0	
	>6.20	8.0	6.8	
Eosinophils*	<0.15	30.8	60.3	0.018
	0.15-0.30	46.2	31.5	
	>0.30	23.1	8.2	
Lymphocytes*	<1.20	4.0	6.8	0.713
	1.20-3.50	96.0	87.7	
	>3.50	0	5.5	
Monocytes*	<0.20	0	1.4	1.000
	0.20-0.80	100	95.9	
	>0.80	0	2.7	
FeNO ppb	<25	61.5	74.0	0.401
	25-50	34.6	21.9	
	>50	3.8	4.1	
ESR mm/h	0-30	100	97.3	1.000
	>30	0	2.7	
Total IgE kU/L	0-100	76.9	86.3	0.351
	>100	23.1	13.7	
CRP mg/L	0-3	88.5	69.9	0.061
	>3	11.5	30.1	

* × 10^9/L
